# Compositional and mutational rate heterogeneity in mitochondrial genomes and its effect on the phylogenetic inferences of Cimicomorpha (Hemiptera: Heteroptera)

**DOI:** 10.1186/s12864-018-4650-9

**Published:** 2018-04-18

**Authors:** Huanhuan Yang, Teng Li, Kai Dang, Wenjun Bu

**Affiliations:** 1 Institute of Entomology, College of Life Sciences, Nankai University, 94 Weijin Road, Tianjin, 300071 China; 2 Institute of Zoology and Developmental Biology, College of Life Sciences, Lanzhou University, 222 Tianshui South Road, Lanzhou, 730000 China

**Keywords:** Mitochondrial genomes, Heterogeneity, Cimicomorpha, Tingidae, Reduviidae, Phylogeny

## Abstract

**Background:**

Mitochondrial genome (mt-genome) data can potentially return artefactual relationships in the higher-level phylogenetic inference of insects due to the biases of accelerated substitution rates and compositional heterogeneity. Previous studies based on mt-genome data alone showed a paraphyly of Cimicomorpha (Insecta, Hemiptera) due to the positions of the families Tingidae and Reduviidae rather than the monophyly that was supported based on morphological characters, morphological and molecular combined data and large scale molecular datasets. Various strategies have been proposed to ameliorate the effects of potential mt-genome biases, including dense taxon sampling, removal of third codon positions or purine-pyrimidine coding and the use of site-heterogeneous models. In this study, we sequenced the mt-genomes of five additional Tingidae species and discussed the compositional and mutational rate heterogeneity in mt-genomes and its effect on the phylogenetic inferences of Cimicomorpha by implementing the bias-reduction strategies mentioned above.

**Results:**

Heterogeneity in nucleotide composition and mutational biases were found in mt protein-coding genes, and the third codon exhibited high levels of saturation. Dense taxon sampling of Tingidae and Reduviidae and the other common strategies mentioned above were insufficient to recover the monophyly of the well-established group Cimicomorpha. When the sites with weak phylogenetic signals in the dataset were removed, the remaining dataset of mt-genomes can support the monophyly of Cimicomorpha; this support demonstrates that mt-genomes possess strong phylogenetic signals for the inference of higher-level phylogeny of this group. Comparison of the ratio of the removal of amino acids for each PCG showed that ATP8 has the highest ratio while CO1 has the lowest. This pattern is largely congruent with the evolutionary rate of 13 PCGs that ATP8 represents the highest evolutionary rate, whereas CO1 appears to be the lowest. Notably, the value of Ka/Ks ratios of all PCGs is less than 1, indicating that these genes are likely evolving under purifying selection.

**Conclusions:**

Our results demonstrate that mt-genomes have sites with strong phylogenetic signals for the inference of higher-level phylogeny of Cimicomorpha. Consequently, bioinformatic approaches to removing sites with weak phylogenetic signals in mt-genome without relying on an a priori tree topology would greatly improve this field.

**Electronic supplementary material:**

The online version of this article (10.1186/s12864-018-4650-9) contains supplementary material, which is available to authorized users.

## Background

The number of complete or nearly complete mitochondrial genomes (mt-genomes) of insects has rapidly increased in recent years with the development of next-generation sequencing technologies [1]. Although widely utilized in phylogenetic analyses, potential biases such as high percentages of AT content, base-compositional heterogeneity between lineages, and high evolutionary rates have all been documented in insect mt-genomes [1–4]. These anomalous characteristics frequently limit their applicability in higher-level phylogenetic reconstruction of insects, leading to an incongruence with morphological and nuclear data [1–4].

These potential biases may lead to systematic errors when the evolutionary model used for phylogenetic inference does not take them into account [3]. Compositional heterogeneity across lineages and sequence saturation due to accelerated substitution rates are two common phenomenon causing phylogenetic artefacts in mt-genome data [5, 6]. The phenomena caused by these potential biases have attracted an increasing amount of attention in studies of some groups, such as Coleoptera, Thysanoptera, Psocodea, Sternorrhyncha (Hemiptera), Strepsiptera, Neuropterida and Hymenoptera [1–3, 7–18], showing that compositional heterogeneity is pervasive. If these characteristics were shared by unrelated lineages, the apparent convergent evolution may erode a genuine phylogenetic signal [6]. Moreover, accelerated evolutionary rates and rate variation across lineages can increase susceptibility to systematic errors, such as long-branch attraction (LBA) [19–21].

Various strategies have been proposed to ameliorate the effects of such biases. For example, first, dense taxon sampling may contribute to inferring multiple substitutions at a site correctly and result in improvements in the estimation of tree topology through improving the estimation of molecular rates and variation in base composition [5, 10, 11, 13, 22]. In addition, the strategy of sampling more taxa to break up long branches has been widely advised to decrease the effects of LBA bias [10, 23–25]. Second, removing the third codon positions from protein-coding genes (PCGs) or using purine-pyrimidine (RY) coding can reduce the effects of compositional heterogeneity and saturation in the assessment of character variation [10, 11, 13, 26–29]. Finally, using evolutionary models such as the site-heterogeneous mixture model [30], an alternative strategy for accommodating complex character variation might mitigate the impact of compositional and mutational bias [3, 10–13, 17, 31–33]. In fact, it has already been shown that the site-heterogeneous mixture model CAT was able to overcome LBA artefacts in some groups [3, 19, 34–37].

**Fig. 1:**
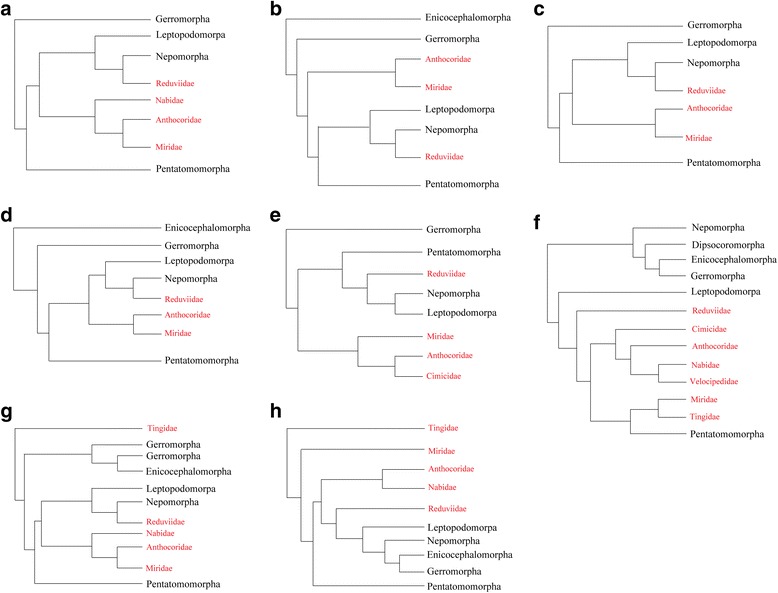
Alternative hypotheses of a paraphyletic Cimicomorpha using mt-genome data. Cimicomorphan species are shown in red. **a** after Li et al. [47]. **b** after Li et al. [48]. **c** after Li et al. [49]. **d** after Li et al. [52]. **e** after Kolokotronis et al. [53]. **f** after Li et al. [54]. **g** after Yang et al. [50]. **h** after Kocher et al. [51]

Cimicomorpha is the largest infraorder in Heteroptera, with 17 families and more than 20,000 species [38]. The monophyly of Cimicomorpha has been consistently and strongly supported by most previous studies based on morphological characters [39–41], molecular data [42–44], and combined data analyses [45], and recently, it was also supported based on the transcriptomic data of 53 taxa and 3102 orthologous genes [46]. However, Cimicomorpha was recovered as a paraphyletic group when the data for mt-genomes alone were used in the analyses [47–54]. The paraphyly of Cimicomorpha was caused mainly by the positions of the families of Reduviidae and Tingidae (Fig. 1). Reduviidae, known as assassin bugs, most of which are predatory insects, is a diverse group of approximately 7000 described species worldwide [55–57]. When the mt-genomes of Reduviidae species were added to the dataset, Reduviidae separated from Cimicomorpha and became the sister group to Nepomorpha (Fig. 1a, b, c, d) or Nepomorpha + Leptopodomorpha (Fig. 1e). In addition, recent studies conducted by Li et al. [54] showed that Reduviidae was supported as the sister group of a clade that included Pentatomomorpha and the remainder of Cimicomorpha (Fig. 1f). Tingidae, known as lace bugs, is a family of approximately 2500 species [58]. When the mt-genomes of a few Tingidae species were used, Tingidae became the sister group to a clade that consisted of the remainder of Heteroptera (Fig. 1g, h), rather than a member of Cimicomorpha. In the above phylogenetic studies recovering a paraphyletic Cimicomorpha, the PCG third codon positions were not removed [47–51, 53], and the analyses were not inferred using heterogeneous models, except in the study by Li et al. [52, 54]; thus, we attempted to incorporate these approaches.

Currently, the mt-genomes of 13 of 18 species of Reduviidae (which belong to 8 subfamilies, 13 genus) reported to date were used in our analyses, while only 4 complete mt-genomes belonging to Tinginae have been sequenced for lace bugs. Here, we sequenced five additional Tingidae species and attempted to discuss the compositional and mutational rate heterogeneity in mt-genomes and its effect on the phylogenetic inferences of Cimicomorpha incorporating the bias-reduction strategies described above.

## Results

### General features of mt-genomes of five newly sequenced species of Tingidae

In this study, we sequenced the mt-genomes of five species of Tingidae (Additional file 1), including one complete (*Trachypeplus jacobsoni*) and four nearly complete (*Cysteochila chiniana*, *Dictyla platyoma*, *Metasalis populi*, and *Tingis cardui*) mt-genomes. In these nearly complete mt-genomes, the control region is incomplete due to highly repetitive regions that could not be assembled with certainty. Each mt-genome contained the typical 37 genes (2 rRNAs, 13 PCGs and 22 tRNAs) with the same gene order observed in the four previously sequenced lace bug mt-genomes [50, 51, 54] and found in most other insects [1]. In the five newly sequenced Tingidae mt-genomes, all PCGs initiated with ATN as the start codon, except for the ATP6 gene, which was only found in *Cysteochila chiniana* and started with GTG. Most PCGs ended with the termination codons TAA/TAG, whereas the remaining PCGs were terminated with T. All typical 22 animal tRNA and 2 rRNA genes were observed in each of these mt-genomes.

### High degree of compositional heterogeneity

**Fig. 2:**
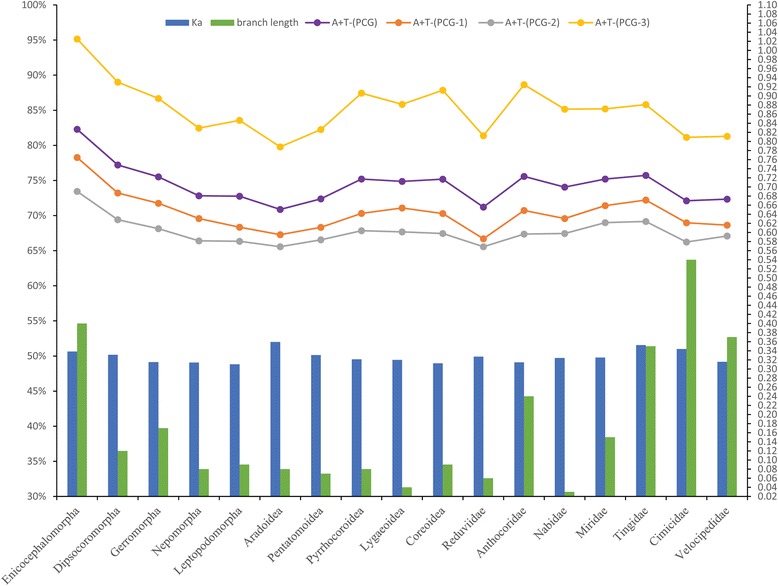
Mean values of base composition, nucleotide substitution rates, and values of branch lengths of each clade were enumerated from each family of Cimicomorpha, each superfamily of Pentatomomorpha and the other five infraorders (Enicocephalomorpha, Dipsocoromorpha, Gerromorpha, Nepomorpha, and Leptopodomorpha). Ka was calculated in a pairwise fashion, using *Abidama producta* as a reference. Estimated branch lengths were extracted from the tree of BI-PCG-gene partition. Although Cimicidae (0.54) and Velocipedidae (0.37) showed rather long branch lengths, these two families both contain only one species, and cannot represent the branch length of a clade

The AT content of PCGs from the included heteropteran species ranged from 65.8% (*Reduvius tenebrosus*) to 83.2% (*Stenopirates* sp.) with a mean of 74.0%. Tingidae PCGs had AT contents between 72.8% (*Cysteochila chiniana*) and 78.2% (*Stephanitis mendica*) with a mean of 75.7% (Fig. 2), which is the highest family-level mean value within the true bugs, except for a few families represented by only one or two taxa. In contrast, the AT content of Reduviidae was between 65.8% (*Reduvius tenebrosus*) and 74.4% (*Oncocephalus breviscutum*) with a mean of 71.2%, which is the lowest family mean of the heteropterans. Analyses of base composition at each codon position across heteropteran insects showed that third codon positions were much higher in AT content than first and second codon positions (Fig. 2).

Individual chi-squared tests of each PCG indicated that all genes were heterogeneous (*P* < 0.05 that the data represented compositional heterogeneity) (Additional file 2). The third codon positions showed significant heterogeneity for all genes (*P* = 0), while all second codon positions appeared homogeneous. The first codon positions revealed heterogeneity (*P* < 0.05) in the genes ND2, ND4, ND5 and ND6, but other PCGs showed compositional homogeneity. When the first and third codon positions were RY recoded and each gene was reanalysed, the first codon positions were no longer apparently heterogeneous in any gene except ND2, but the third positions of most PCGs still remained compositionally heterogeneous in seven genes, including ATP6, CO1, CytB, ND1, ND2, ND4 and ND5 (Additional file 2).

In addition, saturation analyses of each codon position of all PCGs indicated substantial saturation of the substitutions in the third codon positions, while the first and second codon positions were free of saturation (Additional file 3). The AliGROOVE procedure [59] was employed to detect heterogeneous sequence divergence, demonstrating that third codon positions were much more rate-heterogeneous than first and second positions and consistently scored negative in pairwise comparisons (Additional file 4).

### Contrasting rates of evolution in specific lineages

We calculated Ka (non-synonymous substitution rate) for each species in this study using *Abidama producta* (Cercopidae: Auchenorrhyncha) as a reference. In Heteroptera, these comparisons showed that Ka ranged from 0.30 (*Stictopleurus subviridis*) to 0.37 (*Neuroctenus parus*) with a mean of 0.33 (Fig. 2). Notably, Tingidae (0.34–0.36) had the highest family-level mean Ka (0.35) of true bugs, except Aradidae (0.36), which contained only two taxa, while the mean value of Ka (0.33) in Reduviidae was comparable to that of heteropteran insects as a whole. A comparison of branch lengths (Fig. 2) in the phylogenetic tree revealed that Tingidae (0.35) exhibited much longer branch length than the other Heteropteran species except the Enicocephalomorpha (0.40), while Ruduviidae (0.06) had the shortest branch length among Heteropteran species except the Nabidae (0.03) and Lygaeoidea (0.04). Overall, these results indicated contrasting substitution rates among different heteropteran lineages.

### Phylogenetic analyses

#### Dense taxa sampling of Tingidae and Reduviidae

**Fig. 3:**
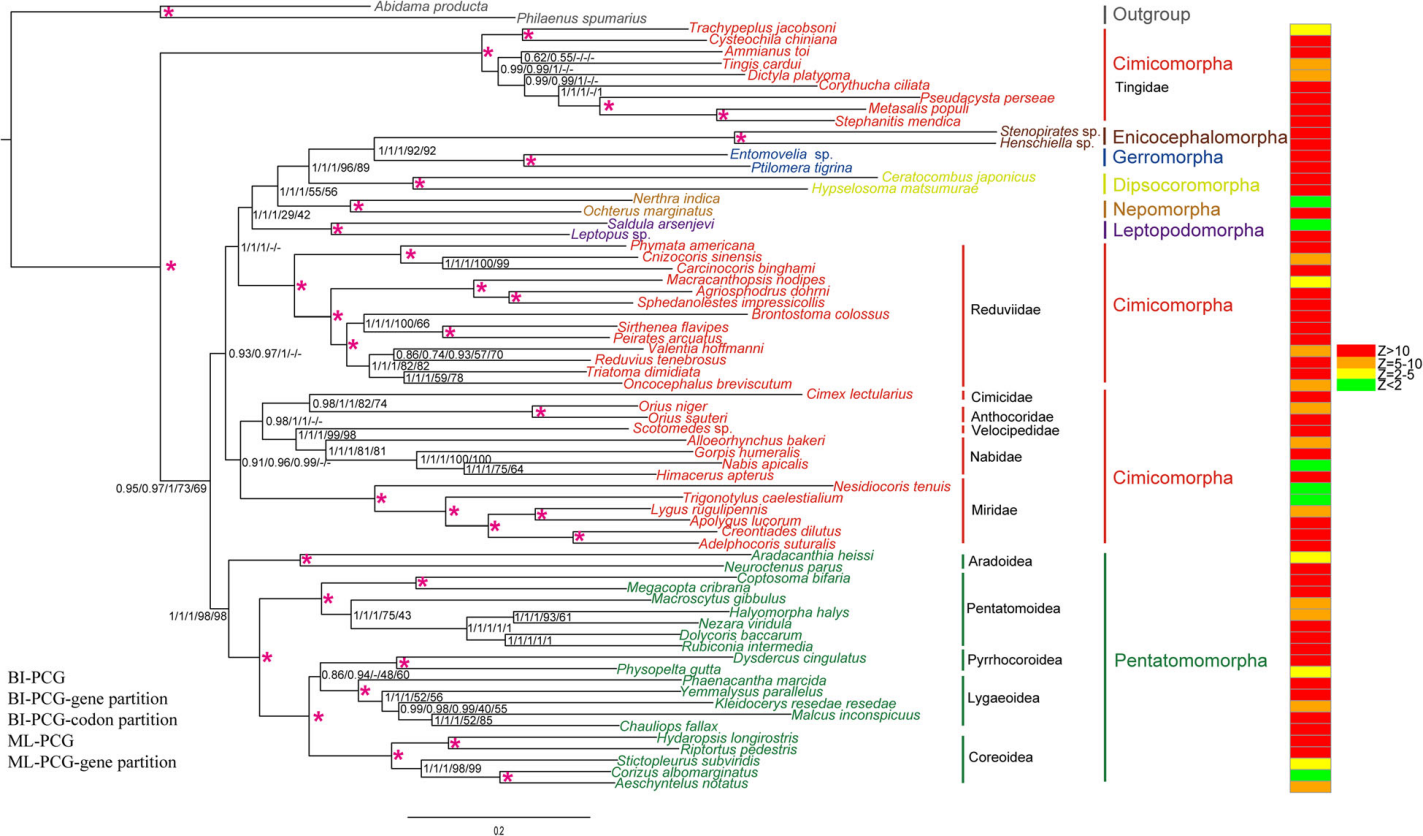
Topologies from analyses of datasets under homogeneous models (BI-PCG, BI-PCG gene partition, BI-PCG-codon partition, ML-PCG, ML-PCG-gene partition). Partitioning schemes and models are listed in Additional file 10. Values at nodes represent Bayesian posterior probabilities (BPP) and ML support values. Asterisks above branches indicate that BPP or ML support values are 1 or 100. A dash is shown if topology is not shown in BI- PCG. Scale bar represents number of expected substitutions per site. Histogram at right is posterior predictive analyses of compositional homogeneity. Z-score > 2 indicated taxa were significantly compositionally heterogeneous

We performed phylogenetic analyses on the mt-genomes of Cimicomorpha with a dense taxon sampling of Tingidae and Reduviidae. Maximum Likelihood (ML) and Bayesian Inference (BI) analyses under homogeneous models indicated that Tingidae was sister to the remaining Heteroptera (Fig. 3), as reported by previous studies based on mt-genomes [50, 51]. A paraphyletic Cimicomorpha was split into three clades Miridae + Cimicidae + Anthocoridae + Velocipedidae + Nabidae, Reduviidae (sister to remaining heteropteran infraorders except Pentatomomorpha and the remainder Cimicomorpha), and Tingidae (sister to remaining heteroptera), which is incongruent with previous studies based on morphological characteristics [39–41] and/or molecular data [42–46]. In addition, BI and ML analyses of an optimized partition scheme (PCG-gene partition and PCG-codon partition) also found a paraphyletic Cimicomorpha (Fig. 3). All these analyses indicated that dense taxon sampling alone did not resolve the problem of artefactual paraphyly of Cimicomorpha.

#### Removal of third codon positions of PCGs or RY coding

**Fig. 4:**
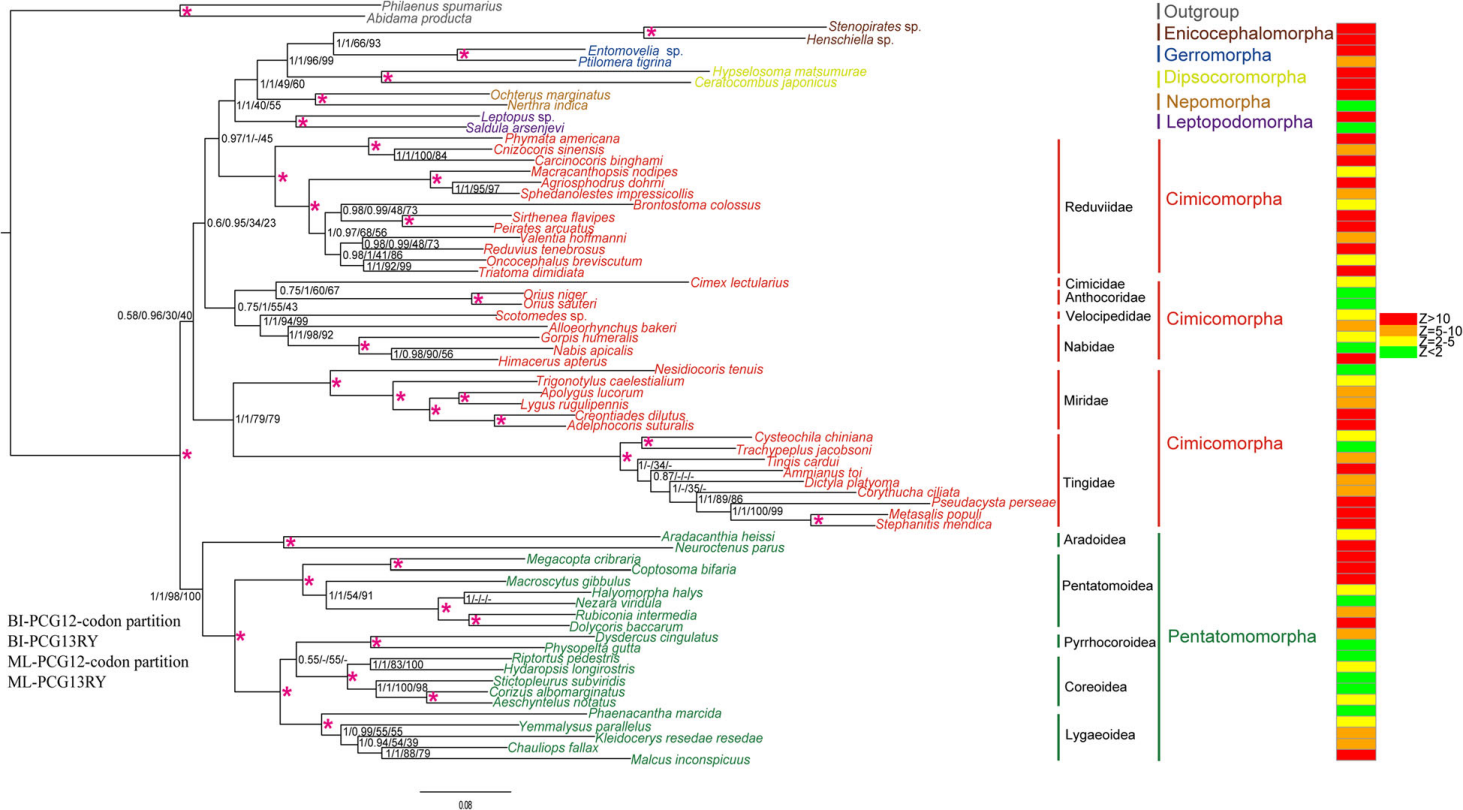
Topologies from analyses of datasets under homogeneous models (BI-PCG12-codon partition, BI-PCG13RY, ML-PCG12-codon partition and ML-PCG13RY). Partitioning schemes and models are listed in Additional file 10. Values at nodes represent BPP and ML support values. Asterisks above branches indicate that BPP or ML support values are 1 or 100. A dash is shown if topology is not shown in BI-PCG12-codon partition. Scale bar represents number of expected substitutions per site. Histogram at right is posterior predictive analyses of compositional homogeneity. Z-score > 2 indicates taxa were significantly compositionally heterogeneous

We excluded the third codon position of PCGs or used RY coding of the first and third codon positions to reduce the effects of compositional heterogeneity. In both BI and ML analyses, removing the third codon positions or used RY coding of first and third codon positions resulted in the monophyly of Miroidea (Tingidae + Miridae) (Fig. 4). The paraphyly of Cimicomorpha changed to three different clades: Reduviidae (sister to the rest of heteroptera, except Pentatomomorpha and the remaining Cimicomorpha), Cimicidae + Anthocoridae + Velocipedidae + Nabidae (sister to remaining heteropteran species except Miroidea and Pentatomomorpha) and Tingidae + Miridae (sister to remaining heteropteran infraorders, except Pentatomomorpha). The monophyly of Reduviidae, Cimicidae, Anthocoridae, Velocipedidae, Nabidae, Tingidae and Miridae were all strongly supported by both Bayesian posterior probabilities (BPP) and bootstrap (BS) values.

Comparing saturation plot slopes estimated by plotting observed distances (uncorrected P-distances) against patristic distances from the CAT + GTR model indicated that the dataset PCG12 showed the lowest level of saturation (PCG12 = 0.0119, PCG = 0.0022 and AA = 0.0104) (Additional file 5).

#### Under heterogeneous models

**Fig. 5:**
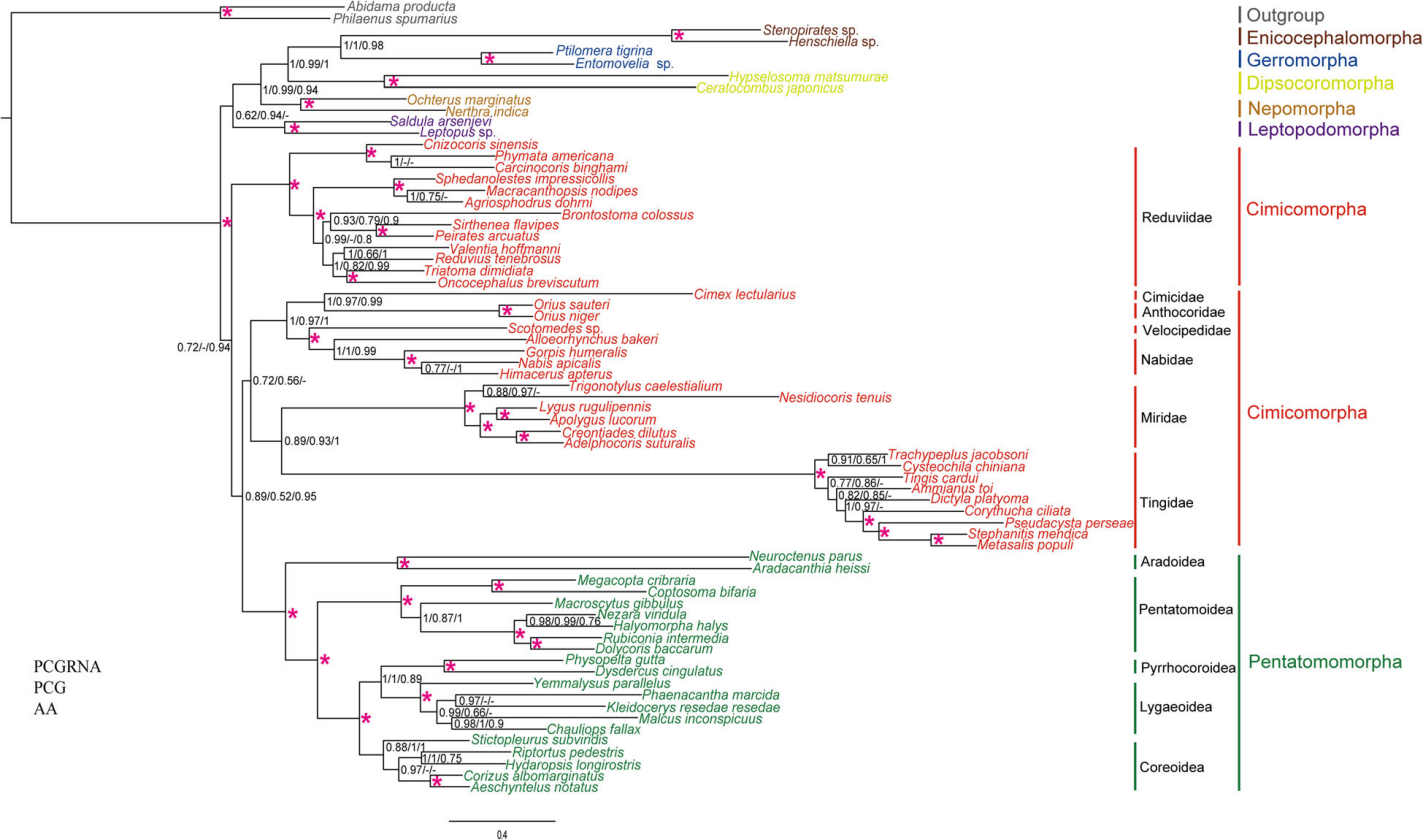
Phylogeny inferred from PhyloBayes analyses under CAT+GTR model with datasets PCGRNA, PCG, and AA. Values at nodes represent BPP. Asterisks above branches indicate support values of 1. A dash is shown if the topology is not shown in PCGRNA. Scale bar represents number of expected substitutions per site

The major changes that resulted from using the CAT + GTR model with three datasets (PCG, PCGRNA and AA) (Fig. 5) were that, although the monophyly of Cimicomorpha was still not recovered, all Cimicomorpha and Pentatomomorpha form a monophyletic group. Cimicomorpha was split into two groups: (Cimicidae + Anthocoridae + Velocipedidae + Nabidae + Tingidae + Miridae) and Reduviidae (sister group of a clade that included Pentatomomorpha and the remainder of Cimicomorpha) (Fig. 5). Moreover, GTR models inferred a much lower level of homoplasy in the nucleotide dataset (PCG, PCG12 and PCGexclude) compared to the CAT + GTR model in posterior predictive analysis (Additional file 6).

#### Removal of sites with weak phylogenetic signals of mt-genomes

Based on the tree topology of Wang et al. [44] in supporting the monophyly of Cimicomorpha, and consistent with most previous studies based on morphological characters, the morphological and molecular combined data and large scale molecular datasets, 4909 sites (497 complete amino acids) with weak phylogenetic signals of PCG dataset were excluded to generate the dataset PCGexclude under a strict filter criterion. The dataset PCGexclude can support the monophyly of Cimicomorpha, which demonstrates that mt-genomes possess strong phylogenetic signals for the inference of higher-level phylogeny of this group. Posterior predictive analyses of compositional homogeneity in PCGexclude indicated that it had the lowest level of heterogeneity, with 45 among 67 species that were still compositionally heterogeneous (Additional file 7), compared to 61 species in PCG (Fig. 3) and 54 species in PCG12 (Fig. 4). The results indicated that removal of sites with weak phylogenetic signals clearly reduced the degrees of heterogeneity.

**Fig. 6:**
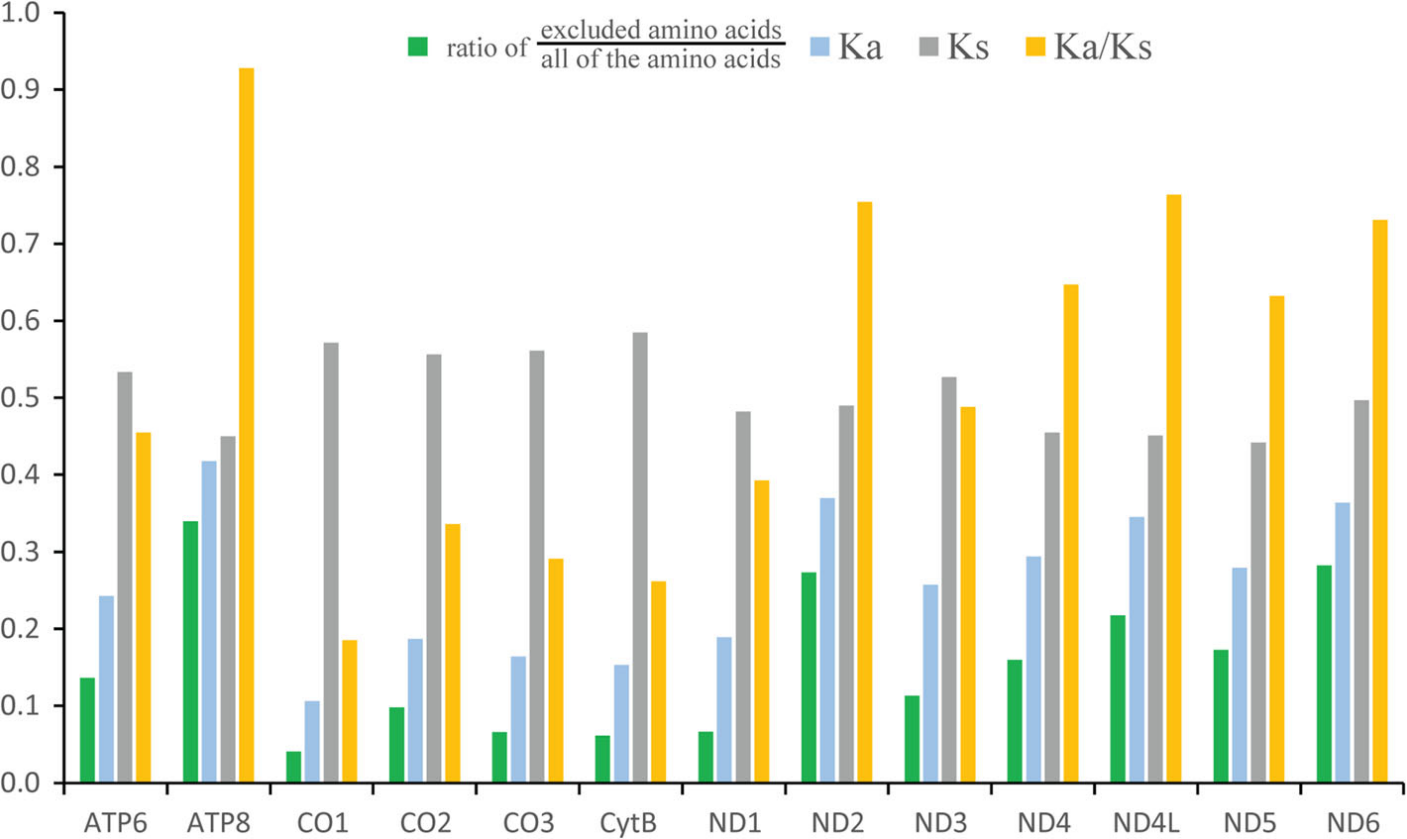
Ratio of excluded amino acids and evolutionary rates of 13 PCGs in Heteroptera. Ratio of excluded amino acids/all amino acids, rate of non-synonymous substitutions (Ka), rate of synonymous substitutions (Ks) and ratio of rate of non-synonymous substitutions to rate of synonymous substitutions (Ka/Ks) were calculated for each PCG

A comparison of the ratio of the excluded amino acids/all 3709 amino acids for each PCG showed that ATP8 has the highest ratio of removed amino acids, while CO1 has the least (Fig. 6). This pattern is largely congruent with the evolutionary rate of 13 PCGs that ATP8 represents the highest evolutionary rate, whereas CO1 appears to be the lowest. The evolutionary patterns of 13 PCGs in our study were consistent with previous studies [48, 52, 60].

## Discussion

### The phylogenetic position of Tingidae and Reduviidae

The monophyly of Cimicomorpha has been consistently supported by studies based on morphological characteristics [39–41], molecular data [42–44] and combined data analyses [45]. Recently, it has also been supported by transcriptomic data of 53 taxa and 3102 orthologous genes [46]. In contrast, phylogenetic analyses based on concatenated sequences of mt-genes always failed to recover Cimicomorpha as a monophyletic group [47–54], caused mainly by the positions of the families of Reduviidae and Tingidae, especially Tingidae, which was placed as the sister group to all remaining heteropterans by Yang et al. [50] and Kocher et al. [51]. These surprising phylogenetic results have no support from morphological data or nuclear gene sequences. We hypothesized that the findings of Yang et al. [50] and Kocher et al. [51] might be due to biases introduced by inadequate taxon sampling, as only one or two Tingidae mt-genomes were included. Increased taxa sampling can often lead to more accurate phylogenetic inference [5, 10, 11, 13, 22], but increased taxa sampling for Tingidae and Reduviidae in our analyses did not obviously improve the result under homogeneous models. Even using data partitioning, the effect was still limited: Tingidae remained the sister-group of a clade that included the remaining species of Heteroptera (Fig. 3).

Because the dense taxon sampling of Tingidae and Reduviidae in our analyses could not solve this thorny problem, we examined the possibility that these findings resulted from base compositional heterogeneity and accelerated evolutionary rates of mt-genomes implied using inappropriate models [6, 7, 17]. Our analyses under homogeneous models indicated that after the removal of the third codon positions, the Tingidae was recovered as the sister group of Miridae entirely outside of all Cimicomorpha, as previously reported (Fig. 4). RY coding is generally thought to decrease saturation and compositional bias [10, 27], but when RY recoding was used in our analyses, Cimicomorpha was still a paraphyletic group (Fig. 4). After verifying the compositional and mutational heterogeneity of the third codon positions, we suspect that the artefactual phylogenetic result might be due to the use of inappropriate models.

Sophisticated evolutionary models, such as the heterogeneous CAT + GTR model, which account for among-site heterogeneity, can lessen the effects of compositional biases and better reflect the evolutionary process [10, 11, 30]. The use of a heterogeneous model predicted homoplasies more efficiently than homogeneous models in our dataset (Additional file 6). Bayesian analyses using PhyloBayes MPI Version 1.7 [61] for the amino acid and nucleotide datasets using heterogeneous CAT + GTR recovered the monophyly of (Tingidae + Miridae). Although the monophyly of Cimicomorpha was not recovered, as (Reduviidae + (other Cimicomorpha + Pentatomomorpha)) formed a clade, it indeed showed a significant improvement over previous studies [47–54] and recovered the monophyly of Terheteroptera (Cimicomorpha and Pentatomomorpha) based on mt-genome data. Our results confirmed the power of site-heterogeneous mixture models for resolving phylogenetic relationships with Cimicomorpha and showed the significance of adequate model selection. This significance suggests that site-heterogeneous models may be preferable models for phylogenetic reconstruction when using mt-genomes.

Several commonly suggested strategies to reduce sources of systematic bias were used in the phylogenetic inference of Cimicomorpha. Our results demonstrate that these strategies (local dense taxon sampling, removal of third codon positions, RY coding and use of the site-heterogeneous model) were insufficient to recover the monophyly of the Cimicomorpha. The phylogenetic relationships based on the dataset removed the weak phylogenetic sites that were consistent with the results based on large scale dataset (Additional file 7), which indicated that the mt-genomes possess strong phylogenetic signals for the inference of higher-level phylogeny of Cimicomorpha. While these sites with weak phylogenetic signals are only used for the inference of higher-level phylogeny, they may still have strong phylogenetic signals for the inference of lower-level phylogeny, such as the genus or species levels.

### Effect of compositional and mutational rate heterogeneity

A high degree of compositional heterogeneity across all PCGs was found in our dataset, with the third codon position showing significant levels of compositional heterogeneity and to a lesser extent the first and second positions, even after RY coding (Additional file 2). Moreover, ND2, 4, 5, and 6 genes are more compositionally heterogeneous than the other PCGs (Additional file 2), and this phenomenon was also observed in a detailed analysis of Coleoptera [10]. This phenomenon-that heterogeneity mainly affected the NADH genes, which are associated with functionality-might indicate that the compositional heterogeneity of the NADH genes is driven by variation in the protein level and possible covariation in the NADH protein complex [10].

Accelerated substitution rates may also play a role in eroding phylogenetic signal through unrecognized homoplasy and can lead to problems with LBA [25, 62, 63]. Species of the Aradidae and Tingidae genera had a markedly accelerated evolutionary rate (Fig. 2). Generally, both species of the two families are weakly flying species. Mitochondria, via oxidative phosphorylation, supply most of the energy required for locomotion, and the metabolic power required for locomotion has a linear correlation with speed [64]; we speculate that with the degeneration of locomotive ability, weakly flying or flightless species might require less energy efficient metabolism than rapidly flying taxa. Thus, the functional constraints are relaxed on the mt-genomes, which might accumulate more nucleotide substitutions [65].

### Removal of sites with weak phylogenetic signals

In our analyses, 4909 sites (including 497 complete amino acids) with weak phylogenetic signals were excluded from 11,127 sites (3709 amino acids) of PCGs. The comparison of the ratio of the excluded amino acids/all 3709 amino acids for each PCG showed that ATP8 has the most amino acids removed, while CO1 has the least; this finding is consistent with the pattern of the evolutionary rate of 13 PCGs, in which ATP8 represents the highest evolutionary rate and CO1 appears to be the lowest (Fig. 6). Notably, the value of Ka/Ks ratios of all PCGs are less than 1, indicating that these genes are likely evolving under purifying selection [52, 66]. In the evolution of mt-genomes, purifying selection is the predominant force [67, 68]. It is possible that when lifestyle changes with greater energy demands or reduced oxygen availability, in this context of strong purifying selection, weak and/or episodic positive selection occurs [67, 68]. The evolutionary patterns of 13 PCGs in our study were consistent with previous studies [48, 52, 60]. As the proteins encoded by mt-genomes provide up to 95% of the cell’s energy requirements, they play a critical role in oxidative phosphorylation [65]. Nonsynonymous substitutions can cause defects in respiratory-chain activity that reduce the efficiency of metabolic processes and are generally more harmful [69, 70]. To maintain functional requirements, the amino acids of CO1 experienced strong evolutionary constraints [71, 72] and undergo strong evolutionary pressures. While ATP8 underwent weak evolutionary pressures and functional constraints, this relaxation of metabolic constraint may enable the accumulation of more mutations in the mt-genomes.

## Conclusions

Our results indicate that it is a challenge to reconstruct the phylogenetic relationships of Heteroptera based on mt-genomes, especially for Cimicomorpha, due to its high degree of compositional heterogeneity and significantly accelerated evolutionary rates in specific lineages. When the strategies of the appropriate model were selected, such as site-heterogeneous mixture models, the monophyly of Terheteroptera (Cimicomorpha and Pentatomomorpha) based on mt-genome data was recovered. Unfortunately, these suggestions cannot completely remove the potential biases in this group, complicating the inference of higher-level phylogeny of Cimicomorpha.

The removal of sites with weak phylogenetic signals from the mt-genomes dataset based on a parsimony-based approach—which strongly relies on a previous generally accepted tree topology—can reduce these potential biases significantly. The phylogenetic relationships of Cimicomorpha based on the dataset, which removed the sites with weak phylogenetic signals, were consistent with the results based on various datasets in previous studies and demonstrated that mt-genomes possess strong phylogenetic signals for the inference of higher-level phylogeny. This analysis can measure how much noisy data must be removed to reduce the impact of weak phylogenetic signal, and we can also determine the source of the noise sites, including particular genes and particular amino-acid residues. The problem will be how to identify sites with weak phylogenetic signals in mt-genome datasets without relying on the topology of an a priori tree. Consequently, bioinformaticians will need to propose new approaches without an a priori tree to uncover these sites in mt-genome datasets, which will greatly improve this field.

## Methods

### Taxa sampling and sequencing

In total, 67 species of Heteroptera, representing all seven infraorders of Heteroptera, were included, with two species of Auchenorrhyncha (*Philaenus spumarius* and *Abidama producta*) as outgroups, as Cicadomorpha was strongly supported as the sister group of Heteroptera in a previous study based on mt-genomes [73]. The mt-genomes of five lace bug species, *Trachypeplus jacobsoni*, *Cysteochila chiniana*, *Dictyla platyoma*, *Metasalis populi*, and *Tingis cardui,* are reported here for the first time. The remainder were retrieved from GenBank, including 24 mt-genomes published previously by our group [25, 44, 49, 74]. Detailed taxon information is included in Additional files 8 and 9.

For the newly sequenced species, total genomic DNA was extracted from thoracic muscle tissue using a CTAB-based method [75]. Then, the entire mt-genome was obtained using the Illumina HiSeq 2000 platform (Illumina, San Diego, CA) with a 200-bp insert size and a paired-end 100-bp sequencing strategy at BGI-Shenzhen, China. The identification of PCGs and ribosomal RNA genes (rRNAs) was performed as in previous studies [52]. The annotation of five newly sequenced mt-genomes of Tingidae is provided in Additional file 1.

### Sequence alignment and dataset concatenation

The sequences of 13 PCGs and 2 rRNAs from mt-genomes were used in our analyses. The methods for the alignment of PCGs and rRNAs were same as in previous studies [52]. Alignments of individual genes were concatenated to generate various datasets to reconstruct the phylogeny: 1) PCG: all three codon positions of the 13 PCGs; 2) PCGRNA: all three codon positions of the 13 PCGs and two rRNAs; 3) AA: amino acid sequences of 13 PCGs. Furthermore, we used PartitionFinder 2.0 [76] to test the various partitioning schemes for ML and BI methods, and the input configuration files, which contained different predefined partitions for each dataset, were created: 1) PCG-gene partition: 13 gene partitions for PCGs; 2) PCG-codon partition: 39 codon partitions for PCGs; 3) PCG12-codon partition: 26 codon positions for the first and second codon positions. We used the Bayesian information criterion (BIC) and the “greedy” algorithm with branch lengths estimated as “unlinked” to search for the best-fit scheme and substitution model (Additional file 10). To decrease saturation and compositional bias, we also used RY coding [26, 27, 29] datasets, as PCG13-RY (PCGs with the first and third codon positions RY coded) was used.

### Compositional heterogeneous and contrasting rates analyses

Base composition was analysed with MEGA 6.0 [77]. We used the chi-squared statistic to test the compositional heterogeneity of PCGs as described in Foster (2004) [78]. Analysis of heterogeneity was conducted in PAUP*4 [79], including the ingroup only. Based on tail area probabilities (Pt), < 0.05 was deemed to indicate compositional heterogeneity. To test whether the taxa in our dataset are compositionally heterogeneous, we conducted posterior predictive analysis under the CAT+GTR model using PhyloBayesv4.1c [31]. A Z-score value of more than 2 indicated that the taxa were significantly compositionally heterogeneous. In addition, AliGROOVE [59] was used to analyse sequence divergence heterogeneity with the default sliding window size. Ambiguity was set for the nucleotide dataset to generate profiles of pairwise sequence similarity for all pairwise sequence comparisons. To test for substitution saturation, we plotted each codon position based on the K80 model for transition and transversion substitutions in DAMBE V4.5.32 [80].

The rate of non-synonymous substitutions (Ka), synonymous substitutions (Ks) and the ratio of Ka/Ks were calculated for each PCG in DnaSP 5.0 [81]. Estimated branch lengths were extracted from the tree BI-PCG-gene partition. Branch lengths of each clade, i.e., each family of Cimicomorpha, each superfamily of Pentatomomorpha and the five other infraorders (Enicocephalomorpha, Dipsocoromorpha, Gerromorpha, Nepomorpha, and Leptopodomorpha) were enumerated. As Cimicidae and Velocipedidae both contain only one species, they cannot represent the branch length of a clade.

### Model-based saturation plots and posterior predictive analyses

To measure how well the models anticipated sequence saturation and homoplasy, we conducted saturation plots and posterior predictive analyses. The best-fit CAT+GTR model was selected as a reference for saturation plots. Observed distances (uncorrected P-distances) and patristic distances generated by PATRISTIC [82] were then calculated, and the regression from the ordered pairs of distances plotted against each other. The slope of the regression line, indicates the level of saturation: the shallower the slope, the greater the level of saturation. PhyloBayes v4.1c [31] was used to conduct posterior predictive analyses to compare alternative models’ ability to estimate homoplasy in our datasets.

### Phylogenetic analyses

#### Using homogeneous models

Phylogenetic analyses were initially conducted using standard BI and ML analyses with homogeneous models. The datasets were not partitioned, and Modeltest 3.7 [83] was used to infer the best substitution models for nucleotide data. BI analyses were conducted using GPU MrBayes [84]. In BI, the GTR + I + G substitution model for nucleotide data was used. Two simultaneous runs with four chains of 10,000,000 generations were conducted for the matrix. Each set was sampled every 1000 generations with a burn-in of 25%. The two runs converged satisfactorily, with a standard deviation of split frequency lower than 0.01, and the effective sample size (ESS) was above 200. ML analyses were conducted using RAxML 8.0.12 [85] with the GTR + I + G model for nucleotide data. Nodal support was calculated with bootstrap values from heuristic searches of 1000 resampled datasets using the rapid bootstrap feature (random seed value 12,345) [86].

#### Using heterogeneous models

For both AA and nucleotide datasets, PhyloBayes MPI Version 1.7 [61] was used to conduct phylogenetic analyses with the CAT+GTR model. Two independent searches were run until the likelihoods stabilized and the two runs had satisfactorily converged (maxdiff less than 0.3). The initial trees of each run were discarded as burn-in, and a consensus tree was computed from the remaining trees.

#### Removing sites with weak phylogenetic signals of mt-genomes

To test whether the dataset of mt-genomes have sites with strong phylogenetic signals for phylogenetic inference at higher-level category of Cimicomorpha, we adopted a parsimony-based approach to detect the weak phylogenetic signals of mt-genomes [87]. The PCG source dataset and the corresponding tree topology from Wang et al. [44] were analysed in PAUP*4.0b10 [79], using DELTRAN optimization. All sequences of the dataset were depicted on the preset branches of the lineages. A labelled tree with a complete list of sites was obtained by activating the log-file options (Describetrees/root = outgroup, plot = phylogram, labelnode = yes, apolist = yes). The consistency index (CI) of each site was used as a filter criterion. It is generally recognized that when the CI value of each site was lower than 0.3, this site was likely probably caused by homoplasy, which means with weak phylogenetic signal [88]. We selected CI (0.3) to filter the sites obtained in the parsimony-based analyses through a series of in-house shell scripts (Additional file 11). Finally, the generated sub-dataset (PCGexclude) excluded the sites with CI values below 0.3 for subsequent analyses.

## Additional files

Additional file 1: Annotation and organization of five Tingidae mt-genomes sequenced in this study. (XLSX 19 kb)

Additional file 2: Conventional chi-squared test of each gene and dataset with each codon position. *P* < 0.05 indicated heterogeneity. PCG1, the first codon position of PCG. PCG2, the second codon position of PCG. PCG3, the third codon position of PCG. PCGRY1, the first codon position was RY recoded. PCGRY3, the third codon position was RY recoded. (TIFF 502 kb)

Additional file 3: Substitution patterns of all codon positions. The number of transition (S) and transversion (V) substitutions are plotted against Kimura 2-parameter (K2p) distance, considering all sites. Each point represents pairwise comparison among two taxa. (TIFF 730 kb)

Additional file 4: Heterogeneous sequence divergence within heteropteran mt-genomes. The mean similarity score between sequences was represented by a coloured square. Scores range from − 1 (indicating a maximally random level of similarity), to + 1 (indicating maximally non-random similarity). Darker red indicates more randomized accordance between the pairwise sequence comparisons. Blue indicates a less randomized accordance. The dataset name of each codon position is listed on the bottom left corner. (TIFF 2403 kb)

Additional file 5: Model-based saturation plots for amino acid and nucleotide datasets. Plots of patristic distances of datasets (PCG, AA and PCG12) as estimated from the CAT+GTR tree, compared to distances estimated from the observed distances (uncorrected P-distances). (TIFF 459 kb)

Additional file 6: Posterior predictive analyses of sequence homoplasy. DH is the difference between the observed and predicted homoplasy. Values closer to zero indicate better predictions. (XLSX 10 kb)

Additional file 7: Topology based on analyses of dataset PCGexclude under homogeneous models. We show a schematic version of the phylogenetic trees, with some lineages collapsed for clarity. Values at nodes represent BPP and ML support values. Asterisks above the branches indicate that BPP or ML support values are 1 or 100. The scale bar represents the number of expected substitutions per site. The histogram on the right was the posterior predictive analyses of compositional homogeneity. A Z-score > 2 indicated taxa were significantly compositionally heterogeneous. (TIFF 1528 kb)

Additional file 8: Collection information of 5 species newly sequenced in present study. (XLSX 9 kb)

Additional file 9: Complete or nearly complete mt-genomes used in this study. Mt-genome sequences of 5 newly sequenced species and 24 species generated from our previous studies were highlighted in bold. (XLSX 15 kb)

Additional file 10: Optimal partitioning schemes selected by PartitionFinder for each dataset. (XLSX 10 kb)

Additional file 11: A series of in-house shell scripts used in this study. (TXT 664 bytes)

## Abbreviations

BI: Bayesian inference
BIC: Bayesian information criterion
BPP: Bayesian posterior probabilities
BS: Bootstrap
ESS: Effective sample size
LBA: Long-branch attraction
MCMC: Markov Chain Monte Carlo
ML: Maximum likelihood
Mt-genome: Mitochondrial genome
NADH: Nicotinamide adenine dinucleotide
PCGs: Protein-coding genes
RY: Purine-pyrimidine
