# Supplementary figures and images for: Compositional and mutational rate heterogeneity in mitochondrial genomes and its effect on the phylogenetic inferences of Cimicomorpha (Hemiptera: Heteroptera)
Source: BMC Genomics. 2018 Apr 18;19:264. doi: 10.1186/s12864-018-4650-9 (PMC5907366; doi:10.1186/s12864-018-4650-9)

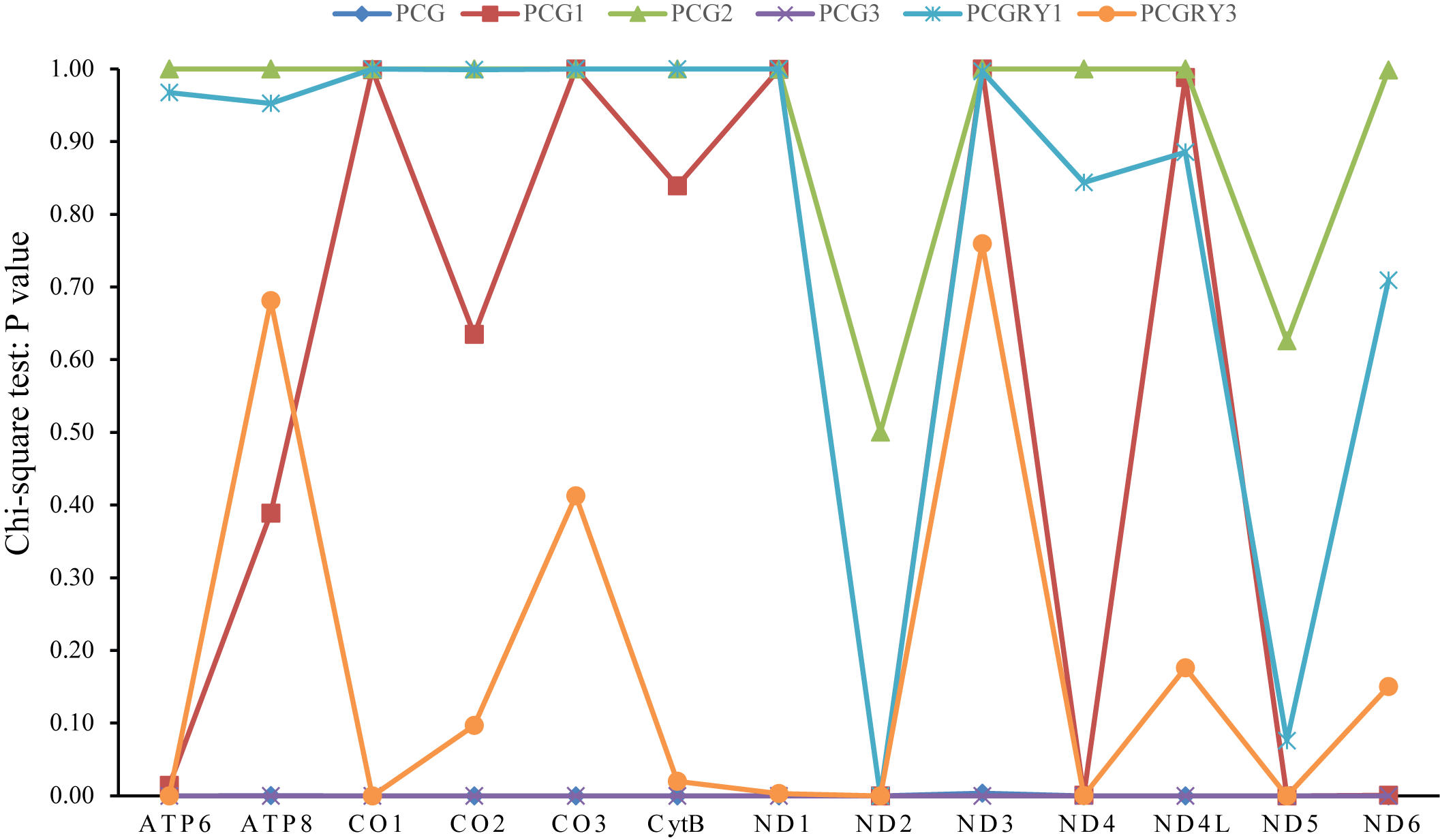

Supplement: Supplementary file 2 — Conventional chi-squared test of each gene and dataset with each codon position. P < 0.05 indicated heterogeneity. PCG1, the first codon position of PCG. PCG2, the second codon position of PCG. PCG3, the third codon position of PCG. PCGRY1, the first codon position was RY recoded. PCGRY3, the third codon position was RY recoded. (TIFF 502 kb) [file 12864_2018_4650_MOESM2_ESM.tif]

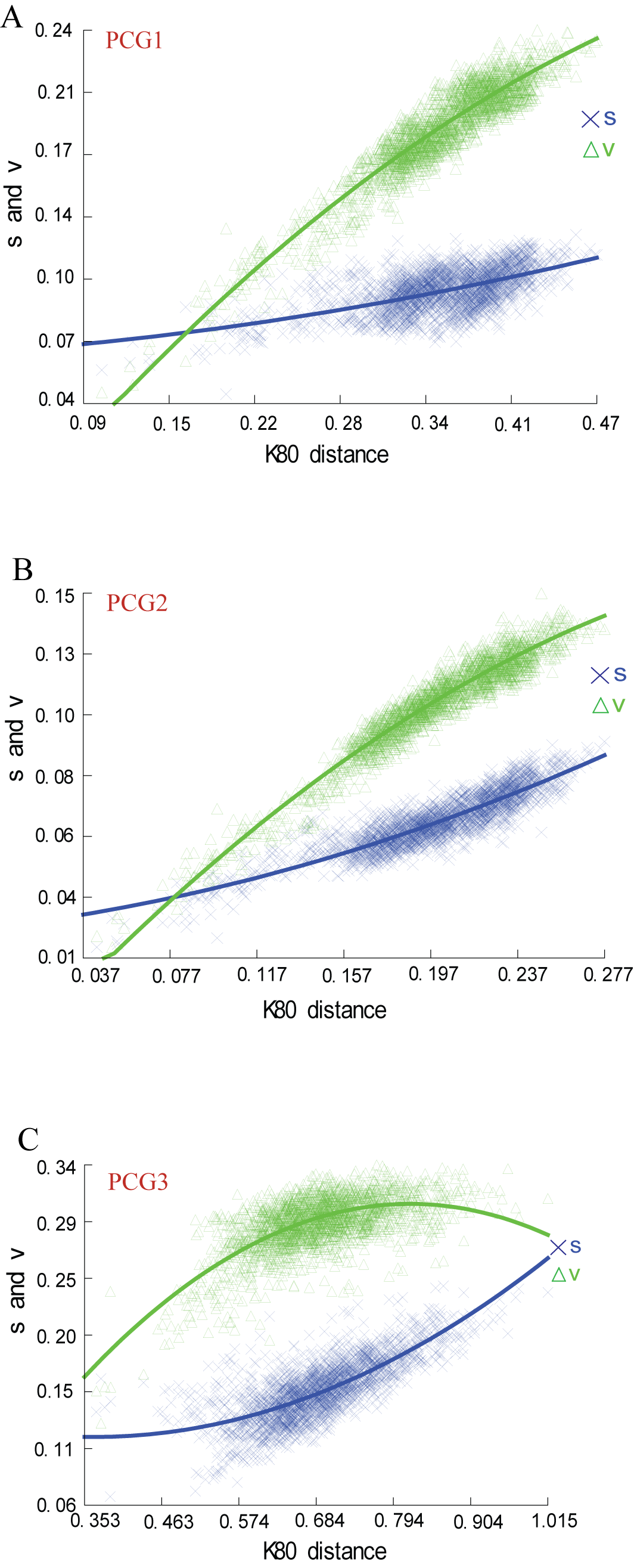

Supplement: Supplementary file 3 — Substitution patterns of all codon positions. The number of transition (S) and transversion (V) substitutions are plotted against Kimura 2-parameter (K2p) distance, considering all sites. Each point represents pairwise comparison among two taxa. (TIFF 730 kb) [file 12864_2018_4650_MOESM3_ESM.tif]

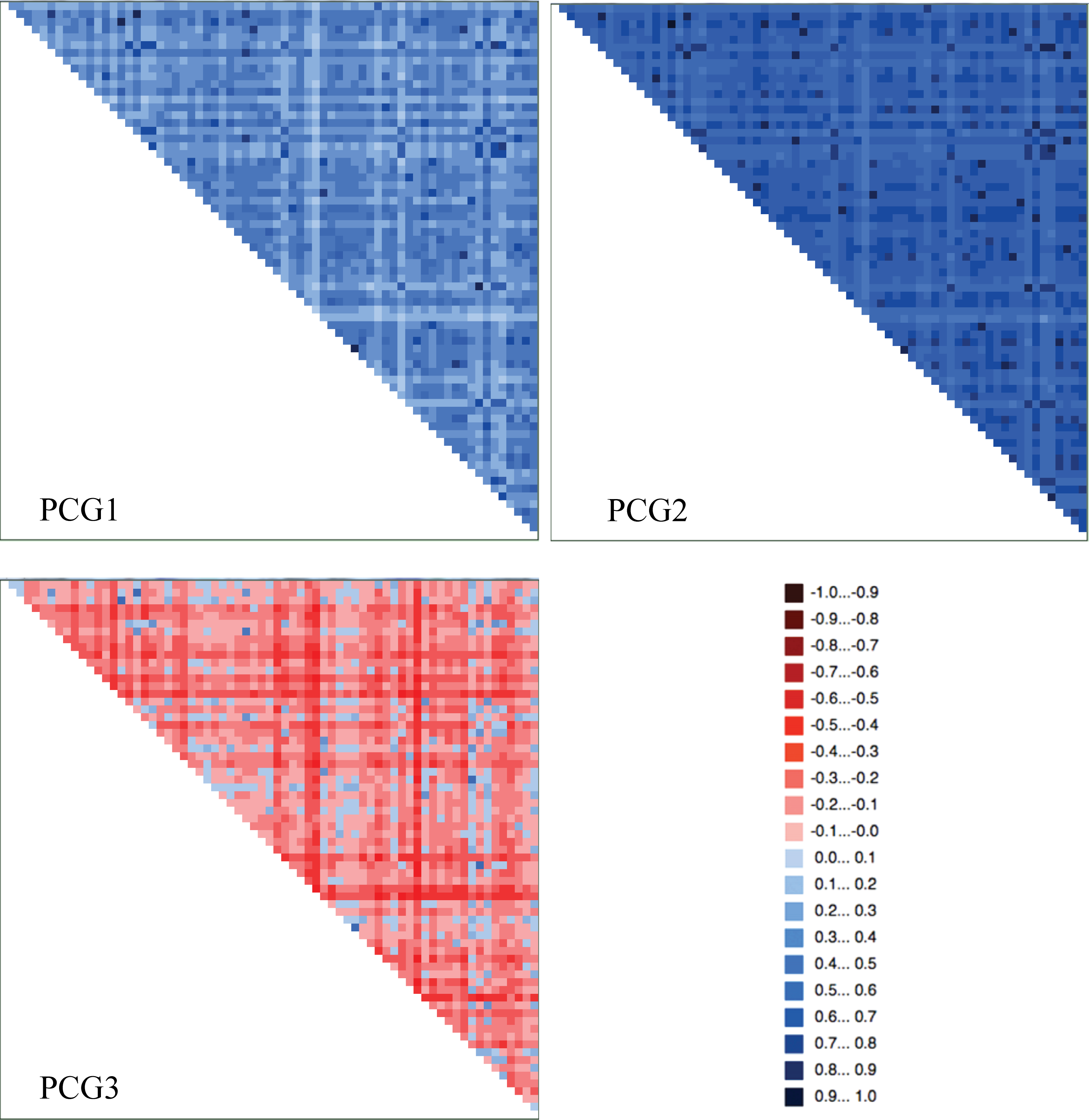

Supplement: Supplementary file 4 — Heterogeneous sequence divergence within heteropteran mt-genomes. The mean similarity score between sequences was represented by a coloured square. Scores range from − 1 (indicating a maximally random level of similarity), to + 1 (indicating maximally non-random similarity). Darker red indicates more randomized accordance between the pairwise sequence comparisons. Blue indicates a less randomized accordance. The dataset name of each codon position is listed on the bottom left corner. (TIFF 2403 kb) [file 12864_2018_4650_MOESM4_ESM.tif]

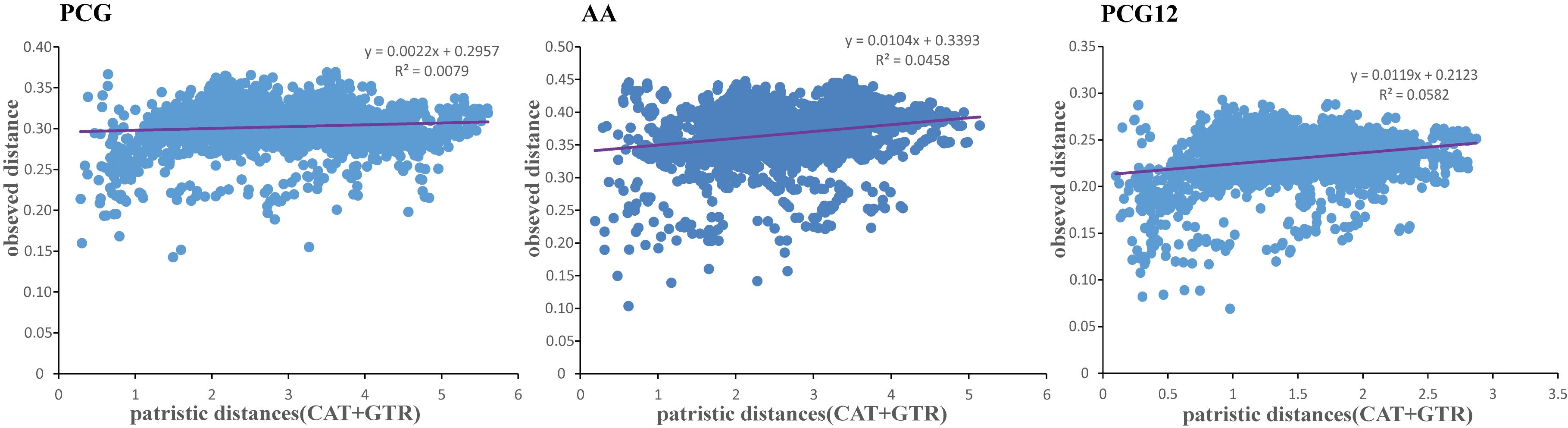

Supplement: Supplementary file 5 — Model-based saturation plots for amino acid and nucleotide datasets. Plots of patristic distances of datasets (PCG, AA and PCG12) as estimated from the CAT+GTR tree, compared to distances estimated from the observed distances (uncorrected P-distances). (TIFF 459 kb) [file 12864_2018_4650_MOESM5_ESM.tif]

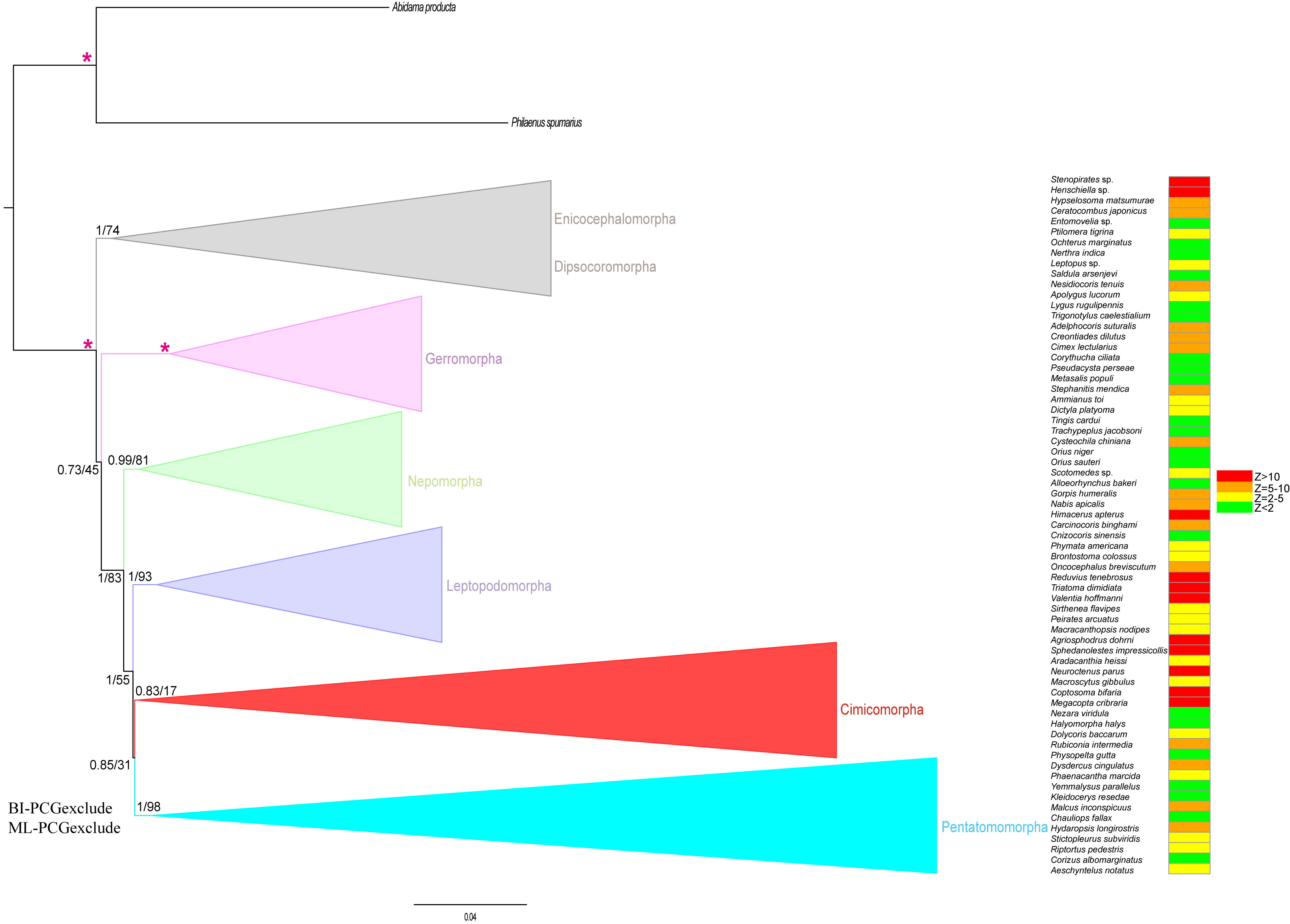

Supplement: Supplementary file 7 — Topology based on analyses of dataset PCGexclude under homogeneous models. We show a schematic version of the phylogenetic trees, with some lineages collapsed for clarity. Values at nodes represent BPP and ML support values. Asterisks above the branches indicate that BPP or ML support values are 1 or 100. The scale bar represents the number of expected substitutions per site. The histogram on the right was the posterior predictive analyses of compositional homogeneity. A Z-score > 2 indicated taxa were significantly compositionally heterogeneous. (TIFF 1528 kb) [file 12864_2018_4650_MOESM7_ESM.tif]
